# The role of pyroptosis in cancer: pro-cancer or pro-“host”?

**DOI:** 10.1038/s41419-019-1883-8

**Published:** 2019-09-09

**Authors:** Xiaojing Xia, Xin Wang, Zhe Cheng, Wanhai Qin, Liancheng Lei, Jinqing Jiang, Jianhe Hu

**Affiliations:** 1 College of Animal Science and Veterinary Medicine, Henan Institute of Science and Technology, Xinxiang, China; 2 College of Agriculture and Forestry Science, Linyi University, Linyi, China; 3 Amsterdam UMC, University of Amsterdam, Center for Experimental and Molecular Medicine, Amsterdam Infection and Immunity, Meibergdreef 9, 1105AZ Amsterdam, Netherlands; 4 College of Veterinary Medicine, Jilin University, Changchun, China

**Keywords:** Cancer, Cancer

## Abstract

Programmed cell death (PCD) refers to the way in which cells die depending on specific genes encoding signals or activities. Apoptosis, autophagy, and pyroptosis are all mechanisms of PCD. Among these mechanisms, pyroptosis is mediated by the gasdermin family, accompanied by inflammatory and immune responses. The relationship between pyroptosis and cancer is complex, and the effects of pyroptosis on cancer vary in different tissues and genetic backgrounds. On one hand, pyroptosis can inhibit the occurrence and development of tumors; on the other hand, as a type of proinflammatory death, pyroptosis can form a suitable microenvironment for tumor cell growth and thus promote tumor growth. In addition, the induction of tumor pyroptosis is also considered a potential cancer treatment strategy. Studies have shown that DFNA5 (nonsyndromic hearing impairment protein 5)/GSDME (Gasdermin-E) mRNA methylation results in lower expression levels of DFNA5/GSDME in most tumor cells than in normal cells, making it difficult to activate the pyroptosis in most tumor cells. During the treatment of malignant tumors, appropriate chemotherapeutic drugs can be selected according to the expression levels of DFNA5/GSDME, which can be upregulated in tumor cells, thereby increasing the sensitivity to chemotherapeutic drugs and reducing drug resistance. Therefore, induced pyroptosis may play a predominant role in the treatment of cancer. Here, we review the latest research on the anti- and protumor effects of pyroptosis and its potential applications in cancer treatment.

## Facts

1. Pyroptosis, a lytic, inflammatory type of regulated cell death that requires membrane-damaging gasdermin proteins, characterized by the swelling and lysis of cells, and release of many proinflammatory factors.

2. The inflammasome, caspase and gasdermin family are play key roles in pyroptosis.

3. Pyroptosis, its associated signaling pathways and the release of various inflammatory mediators are closely related to the tumorigenesis and drug resistance of tumors.

4. Triggering tumor (especially apoptosis resistance) pyroptosis holds great therapeutic potential for cancer treatment.

## Open questions

1. Does pyroptosis play differential roles in normal and tumor tissues?

2. What are the key signals that initiate pyroptosis?

3. What are the key signaling pathways impacted by pyroptosis in tumors?

4. How can pyroptosis be manipulated to drive tumor fate?

## Introduction

The dynamic balance between cell proliferation, differentiation and death maintains ontogeny, homeostasis and pathological processes in multicellular organisms. Cell death are mainly divided into two categories, necrosis and programmed cell death (PCD). Apoptosis is a type of PCD involving the automatic self-destruction of cells controlled by genes, the cell membrane remains intact, and generally not inducing inflammation. Necrosis is a passive type of cell death caused by pathological stimuli. The cell membrane permeability of necrotic cells increases, causing the cells to swell and eventually breakdown to release the cellular contents, leading to inflammatory reaction^1^. Pyroptosis is a new procedural and inflammatory death discovered after apoptosis and necrosis. Similar to apoptosis, pyroptotic cells undergo nuclear condensation and chromatin DNA fragmentation, and TUNEL staining is positive^2,3^. Similar to necrosis, during pyroptosis, the formation of the pores disrupts the balance of ion gradients on both sides of the cell membrane, leading to water inflow, cell swelling, cell membrane rupture, and the release of proinflammatory mediators, including IL-1β, IL-18, ATP, and HMGB1^4^, which induce inflammatory responses, thus pyroptosis is also known as inflammatory “necrosis”^5,6^.

A close relationship between pyroptosis and various human diseases, especially malignant tumors. Pyroptosis may play a dual role in the pathogenesis of tumors. On one hand, the multiple signaling pathways and inflammatory mediators released during pyroptosis are closely related to the tumorigenesis as well as to their drug resistance to chemotherapeutic drugs^7–9^. On the other hand, as a type of death, pyroptosis can inhibit the occurrence and development of tumors^7,10^. The role of pyroptosis in tumor has become increasingly prominent as research has advanced. This review will summarize and discuss the potential effects of pyroptosis on cancer and the role of pyroptosis in anticancer therapy.

## Discovery of the cell pyroptosis phenomenon

The term pyroptosis combines the Greek roots ‘pyro’ and ‘ptosis’, which mean fever and falling, respectively, to define a newly discovered inflammatory PCD^11^. As early as 1990s, scientists discovered that *Shigella flexneri* or *Salmonella* infection of mouse macrophages or human monocytes cause cell death^12,13^. In 1997, Arturo Zychlinsky found that *Shigella dysenteriae* could activate caspase-1 in host cells^14^. In 1999, the Arturo Zychlinsky laboratory found that knocking out caspase-1 could block the cell death caused by *Salmonella*^15^. In 2001, the laboratories of Lawrence H. Boise and Brad Cookson gradually elucidated that the macrophage death caused by bacterial infection was a death mode completely different from apoptosis and named it caspase-1-dependent programmed necrosis^11,16^.

**Fig. 1:**
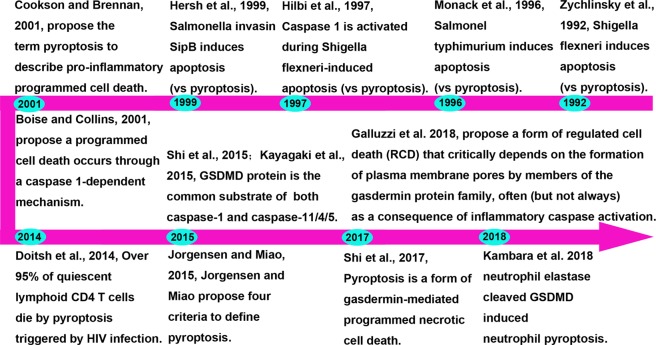
Timeline summary of the history of pyroptosis

But until recently, a new gasdermin-D (GSDMD) protein has been discovered and identified, which normally in a state of auto-inhibition. After caspase cleavage, GSDMD releases the N-terminal fragment (GSDMD-cNT), in turn, the cells swell until they rupture^17,18^, indicating that GSDMD is the executor of pyroptosis. Like GSDMD, other members of the gasdermin family include GSDMA, GSDMB, GSDMC, DFNA5/GSDME, and DFNB59 also have membrane perforation activity and induce pyroptosis^18,19^. Wang et al. confirmed that the N-terminal domain of GSDME combines with 4,5-diphosphate phosphatidylinositol [PI (4,5) P2], leading to the perforation of liposomes and loss of their phospholipid contents, which is consistent with the mechanism of pyroptosis caused by the GSDMD-cNT^20^. Therefore, the Feng Shao group redefined pyroptosis as gasdermin family-mediated programmed necrosis^21^. More recently, Kambara et al. reported that neutrophil elastase (NE) cleaved GSDMD and this cleavage induced neutrophil pyroptosis^22^. In 2018, the Nomenclature Committee on Cell Death (NCCD) proposed defining pyroptosis as a form of regulated cell death (RCD) that critically depends on the formation of plasma membrane pores by members of the gasdermin protein family, often (but not always) as a consequence of inflammatory caspase activation^23^. Figure 1 summarizes the pyroptosis history to date.

## Signaling transduction of pyroptosis

### Inflammasome activation is the basis of pyroptosis

Pattern recognition receptors (PRRs) recognize pathogen-associated molecular patterns (PAMPs) or nonpathogen-related damage-associated molecular patterns (DAMPs), which initiate pyroptosis. Studies have shown that PRRs related to pyroptosis include Toll-like receptors (TLRs), intracellular nucleotide-binding oligomerization domain (NOD)-like receptors (NLRs) and AIM2-like receptors (ALRs)^24^. PRRs recognize PAMPs or DAMPs that are specific to each inflammasome and initiate assembly to recruit and ultimately facilitate caspase-1 dimerization and activation^25,26^. For example, NLRPlb can detect lethal toxins from *Bacillus anthracis* and components of *Toxoplasma gondii*. Many stimulants, such as bacteria, viruses, fungi, uric acid, and ATP, can activate NLRP3 inflammasomes, while flagellin and type III secretory system proteins can be recognized by the NLRC4, and AIM2 inflammasomes primarily recognize double-stranded DNA contained in bacteria or viruses^27^. When PRRs are stimulated, caspase-1 was recruited directly or via ASC to form caspase-1-dependent inflammasome^25,28^. After assembly of the inflammasome, caspase-1 becomes self-activated and changes from a proenzyme to protease, which plays further physiological role.

### Activation mechanism of caspase-1

Caspase-1 exist as a proenzyme in the resting state and is an essential component of the inflammasome, which is formed by different PRRs through (or without) ASC and caspase-1 under the stimulation of specific PAMPs and DAMPs. The inflammasome contains an NLR and the adaptor protein ASC, associating via caspase recruitment domain interactions with procaspase-1, then undergoes autocleavage to form active caspase-1. Caspase-1 not only can mediate the maturation and secretion of proinflammatory cytokines (interleukin-1β (IL-1β) and IL-18) but also initiate the pyroptosis^29^. Joosten et al. found that IL-1 recognizes antigens and induces inflammatory responses. In addition, IL-1 stimulates the activation of primary T cells and memory T cells^30^. IL-18 promotes the synthesis of interferon in Th1 lymphocytes, natural killer cells, and cytotoxic T cells; promotes the differentiation of Th2 cells; and increases local inflammatory responses^31^. Fink et al. found that caspase-1 activation induced the cellular formation of many small pores on the cell membrane. These pores induce communication between the internal and external sides of the membrane, and both sides rapidly lose their ion gradient. Because a large amount of water enters the cell, cell swelling, and the cell eventually dies, allowing the cytoplasmic contents to escape^4^. Therefore, the RCD mediated by caspase-1 is also known as the classical pyroptosis pathway.

### Activation mechanism of caspase-4/5/11

Nonclassical pyroptosis pathways also exist, such as cytoplasmic lipopolysaccharide (LPS) directly activates caspase-4/5/11 to mediate pyroptosis^32^. Caspase-4/5/11 can be directly stimulated by intracellular Gram-negative bacterial LPS to activate and hydrolyze the own protease activity. Activated caspase-4/5/11 can also act on GSDMD and produce the same cleavage effect as caspase-1, leading to the formation of cell membrane pores. Activated caspase-4/5/11 can physically interact with caspase-1 to promote its activation in the presence of NLRP3 and ASC^33–36^. Caspase-1 cleaves precursors of IL-1β and IL-18 to form active IL-1β and IL-18, which can be released through channels formed by GSDMD-cNT and cause pyroptosis^34,36^. Notably, in the nonclassical pyroptosis, only the cleavage of the IL-1β and IL-18 precursors is dependent on caspase-1. For the cleavage of GSDMD, caspase-1 is not required^33^.

### GSDMD is a common substrate for caspase-1/4/5/11

Although the inflammatory caspases initiates the pyroptosis, its specific mechanism is not well understood. Recent studies have found that the GSDMD is a common substrate for caspase-1/4/5/11^17,33,37^, and a highly conserved caspase-1/4/5/11 cleavage site exists in the hinge region of GSDMD^17,33,37–39^. At this cleavage site, if the wild-type “FLTD” sequence is mutated to “LTA”, the cells are no longer able to respond to pedestal proteins and LPS. The overexpression of GSDMD-cNT alone can cause very strong pyroptosis, while the overexpression of the C-terminal domain and full-length GSDMD do not cause pyroptosis^17,18,40^. When activated caspase-1/4/5/11 cleaves the hinge region between the N- and C-terminal domains to produce GSDMD-cNT, the autoinhibition activity of C-terminal domain is relieved, and the lethal activity of the N-terminal domain is released, causing pyroptosis. GSDMD-cNT bind with phosphatidylinositol, phosphatidic acid, and phosphatidylserine on the inner surface of cell membranes and form pores in the lipid bilayer, which is the basis for the interleukins secretion^17,18,40,41^.

### ‘Alternative pathway’ of pyroptosis

**Fig. 2:**
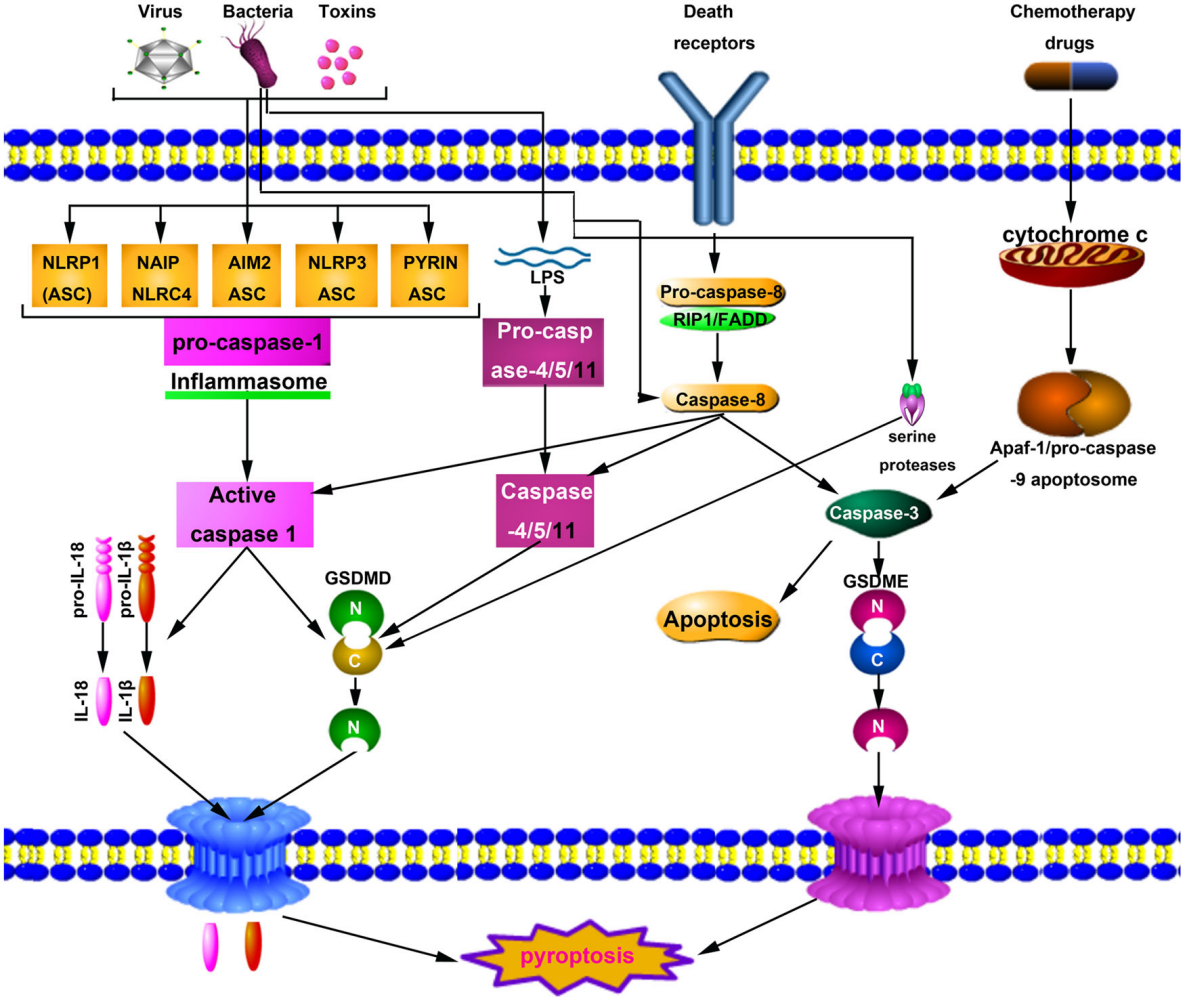
Schematic representation of pyroptosis pathways. The canonical pathway upon sensing DAMPs, PAMPs or other cytosolic disturbances results in the recruitment and activation of caspase-1 either directly or through recruitment of the receptor protein ASC. Caspase-1 successively promotes maturation of the precursors of IL-1β and IL-18 into mature forms and cleaves GSDMD. The pore form domain (PFD) of GSDMD interacts with the plasma membrane to form GSDMD pores, resulting in the release of intracellular contents, including IL-1β and IL-18. The noncanonical pathway is initiated by the caspase-11 self-detection of cytosolic LPS in Gram-negative bacteria. Activated caspase-11 (caspase-4 or caspase-5 in humans) successively cleaves GSDMD and induces pyroptosis. The other pathway of pyroptosis can be engaged through mechanisms such as CASP8-GSDMD and CASP3-GSDME. In turn, activated caspase-3 cleaves GSDME to produce GSDMD-cNT, which forms pores in the plasma membrane and activates pyroptosis. The bacteria are recognized by TLR4, which signals via RIP1 to form a cell death complex consisting of RIP1, caspase-8, and FADD. Both this complex cleavage of GSDMD and activation through caspase 1/11 and GSDME cleavage via caspase-3 lead to cell membrane permeabilization and subsequent pyroptosis. In addition, neutrophil elastase (NE) is able to cleave GSDMD independently of caspase activity

In 2017, Rogers et al. found that activated caspase-3 can cleave DFNA5/GSDME, to generate N-terminal fragment (GSDME-NT) and induce cell pyroptosis after caspase-3 successfully induces apoptosis^42^. Wang et al. also verified the cleavage and activation of GSDME by caspase-3 and further confirmed that the pyroptosis is a mechanism underlying the toxic side effects of some chemotherapeutic drugs^20^. These authors showed that the caspase-1 cleavage site on the GSDMD could be replaced by the caspase-3 cleavage site in HeLa cells. The combined use of TNF-α and cycloheximide activates caspase-3, which further cleaves the GSDMD to produce GSDMD-cNT and induce the apoptosis-to-pyroptosis switch. These authors also found that the expression of wild-type DFNA5/GSDME in HeLa cells reversed caspase-3 activation, causing an apoptosis-to-pyroptosis switch. Wang et al. identified a specific caspase-3 cleavage region between the C- and N-terminal domains of DFNA5/GSDME and established that cells undergo apoptosis after mutation in this cutting region. These researchers further confirmed that the GSDME-NT share the same mechanism caused by GSDMD-cNT^20^. Recent studies have indicated that apoptotic caspase-8 can also induce the cleavage of GSDMD and DFNA5/GSDME, thus inducing pyroptosis during Yersinia infection, which indicates that apoptosis and pyroptosis may share many signal pathways^43–45^. It is interesting to note that serine proteases, including NE and cathepsin G (CatG), can cleave GSDMD independently of caspase activity to generate a fully active NE-derived N-terminal fragment (GSDMD-eNT) and the signature N-terminal domain GSDMD-p30 to induce pyroptosis in neutrophils^22,46^ (Fig. 2).

## Role of pyroptosis in tumorigenesis and metastasis

The tumorigenesis is related to various factors, including the activity of proto- and antioncogenes, the immune microenvironment, oxidative stress and chronic inflammation. The long-term exposure of tissues and/or cells to the inflammatory environment increases the risk of cancer. The activation of pyroptosis leads to the release of the inflammatory mediators IL-1 and IL-18, which could promote the occurrence of cancer in many ways. Studies utilizing *Nlrp3*^−*/*−^ and *caspase-1*^*−/−*^ mice have shown that mice lacking active inflammasomes are more sensitive to azoxymethane/dextran sulfate sodium (AOM/DSS)-induced colitis-associated colon cancer (CAC) than control mice^47–50^. These studies indicated that pyroptosis may play a dual role in promoting and inhibiting tumor cell growth in different tumor cells. However, the specific mechanism of pyroptosis and its rele in tumorigenesis deserve further study.

### Pyroptosis and hepatocellular carcinoma (HCC)

Wei et al. found that the expression of NLRP3 in HCC tissues was significantly downregulated or even completely absent, and its expression was negatively correlated with the pathological grade and clinical stage of HCC, indicating that the NLRP3 inflammasome was involved in the progression of HCC^51^. Furthermore, they found that 17β-estradiol exerted anticancer effects, which attributed to its ability to trigger pyroptosis via activation of NLRP3 inflammasome^52^. The AIM2 inflammasome can weaken the activation of S6K1 by targeting mTOR, thus inhibiting the growth of cancer cells, and the accumulation of the AIM2 inflammasome can cause HCC cells pyroptosis, exerting antitumor effects^53,54^. Caspase-1 was significantly reduced in HCC tissues, and the caspase-1, IL-1β, and IL-18 expression were lower in HCC tissues than these in adjacent normal tissues^55,56^. The expression of *DFNA5/GSDME* in HCC cells is significantly lower than that in normal cells and upregulating *DFNA5/GSDME* expression inhibited cell proliferation, indicated that *DFNA5/GSDME* may be an antioncogene^57^. In addition, the lncRNA CCND2-AS1 involved in improper regulation of pyroptosis in HCC, showing a unique feature of HCC^58^ (Table 1).

**Table 1:** Expression of pyroptosis core proteins in cancer and their impacts on cancer

	Expression level	Tumor type	Prognosis	Ref (s)
NLRP3	Low protein level	Hepatocellular carcinoma (HCC)	NLRP3 deficiency is significantly correlated with advanced stages and poor pathological differentiation.	^51^
AIM2	Low protein level	Hepatocellular carcinoma (HCC)	Low protein level of AIM2 promotes HCC progression.	^53^
Caspase-1	Low protein level	Hepatocellular carcinoma (HCC)	Not determined.	^56^
DFNA5/GSDME	Low protein level	Hepatocellular carcinoma (HCC)	DFNA5 may function as a tumor suppressor gene with an important role in HCC.	^57^
GSDMB	High protein level	Breast cancer (BC)	GSDMB induces invasion, tumor progression and metastasis in MCF7 cells.	^59^
GSDME	Not determined	Breast cancer (BC)	Not determined.	^20^
NLRP3	Low protein level	Colorectal cancer (CRC)	The NLRP3 inflammasome functions as a negative regulator of intestinal tumorigenesis.	^49^
NLRP1	Low protein level	Colorectal cancer (CRC)	The NLRP1 inflammasome functions as a negative regulator of intestinal tumorigenesis.	^68, 69^
AIM2	Low protein level	Colorectal cancer (CRC)	Lack of AIM2 expression is closely associated with poor outcomes in colorectal cancer.	^66^
GSDMA	High protein level	Colorectal cancer (CRC)	GSDMA is overexpressed in carcinoma.	^72^
GSDMC	High protein level	Colorectal cancer (CRC)	GSDMC functions as an oncogene, promoting cell proliferation in colorectal carcinogenesis.	^73^
GSDMD	High protein level	Colorectal cancer (CRC)	GSDMD is downregulated at both the mRNA and protein levels in carcinoma.	^72^
GSDME	High protein level	Colorectal cancer (CRC)	GSDME may be a promising biomarker for the detection of colorectal cancer.	^74^
GSDMC	High protein level	skin cancer	GSDMC plays an important role in promoting proliferation in colorectal tumorigenesis in vivo.	^73^
GSDME	Low protein level	skin cancer	A decreased DNFA5 mRNA expression level is associated with increased etoposide resistance in melanoma cells.	^77^
NLRC4	Low protein level	Gastric cancer (GC)	The NLRC4 expression level in gastric cancer cells is higher than that in normal gastric epithelial cells.	^79^
GSDMA	Low protein level	Gastric cancer (GC)	GSDMA is downregulated in gastric cancer cells and is thought to be a tumor suppressor gene.	^81, 82^
GSDMB	High protein level	Gastric cancer (GC)	GSDMB is increased in gastric cancer cells and is thought to be a tumor suppressor gene.	^82, 89^
GSDMC	Low protein level	Gastric cancer (GC)	GSDMC is downregulated in gastric cancer cells and is thought to be a tumor suppressor gene.	^73, 82^
GSDMD	Low protein level	Gastric cancer (GC)	GSDMD expression is decreased in GC, and the decreased expression of GSDMD could markedly promote the proliferation of tumors in vivo and in vitro.	^82^
GSDME	Low protein level	Gastric cancer (GC)	GSDME may be a tumor suppressor gene.	^73^
GSDMD	High protein level	Lung cancer	High GSDMD expression indicates a poor prognosis in LUAD.	^91^
GSDME	High protein level	Lung cancer	GSDME overexpression leads to enhanced drug sensitivity in vivo and in vitro.	^91^
GSDME	High protein level	esophageal squamous	GSDME is more highly expressed in esophageal squamous cell carcinoma than in normal adjacent tissues.	^104^
GSDML	Unknown	gastric, liver and colon carcinomas	The GSDML protein splicing variants range in molecular weight from 35 to 50 kDa, and the expression profile varies between tumor and nontumor.	^84^

### Pyroptosis and breast cancer (BC)

In BC, a high level of GSDMB is associated with a low survival rate and a high metastasis rate. For HER2-positive BC, overexpression of GSDMB predicts low reactivity to HER2-targeted treatment^59^. Therefore, GSDMB may become a new marker for BC and participate in the evaluation of prognosis. *DFNA5/GSDME*, initially termed ICERE-1, is overexpressed in ER-negative cell lines and may participate in tumorigenesis specific to hormonally unresponsive BC^60^. Interestingly, this methylation was detected in only estrogen receptor-positive cell lines^61,62^. Moreover, *DFNA5* methylation was found to be associated with lymph node metastasis^62^. A study comparing paclitaxel (PTX) drug sensitivity before and after low DFNA5/GSDME expression in MCF-7 cells showed that low DFNA5/GSDME expression reduces the sensitivity of MCF-7 cells to PTX drugs, i.e., the decreased-GSDME increases the resistance of MCF-7 cells to PTX^63^. p53 can induce DFNA5/GSDME expression via a specific p53 binding site in intron 1 of DFNA5^64^. As a member of the p53 family, P63γ also increases DFNA5 levels, suggesting that DFNA5 is a transcriptional target of the p53 family^61^ (Table 1).

### Pyroptosis and intestinal cancer

Studies on *Nlrp3*^*−/−*^ or *caspase-1*^*−/−*^ mice have shown that mice lacking active inflammasomes are more sensitive to AOM/DSS-induced CAC than control mice^47–50^. Study showed that AIM2 inflammasome-mediated pyroptosis plays a key role in radiation-induced gastrointestinal syndrome^65^. Dihlmannd et al. reported that the expression of AIM2 was decreased in 67.4% of colorectal tumors (CRC) cells and absentd in 9.18% of CRC cells. After adjusting for factors such as gender, tumor stage, age, tumor grade, tumor site and chemotherapy, the mortality of 5a patients with *AIM2* deficiency increased^66^. These results indicate that the AIM2 inflammasome is closely related to CRC and/or pyroptosis^65–67^. Studies have reported that the expression of NLRP1 in CRC tissues was decreased compared with normal tissues, and *Nlrp1b*^*−/−*^ mice showed a higher tumor incidence than control mice^68^. The levels of the NLRP1 inflammasome in CRC tissues are lower than those in adjacent tissues. Stage III and IV CAC patients have lower NLRP1 inflammasome than stage I and II CAC patients. Survival analysis have revealed that lower NLRP1 are correlated with a shorter patient survival period^69^. In addition, compared to wild-type (WT) littermates, *Casp11*^*−/−*^ mice are highly vulnerable to the AOM/DSS model of CAC^70^. GSDMA was not expressed in normal colorectal epithelial cells but was gradually overexpressed in carcinoma cells, while GSDMD exhibited the opposite trend^71,72^. *Gsdmc* is not detected in normal colorectal tissues but is present in CRC tissues. Miguchi et al. found that inactivation mutations of *Tgfbr2* often occur, which upregulates *Gsdmc* expression, induces tumor cell proliferation and promotes tumorigenesis. Therefore, *Gsdmc* is an oncogene that may act as a new therapeutic target for CRC treatment with the Tgfbr2 mutation^73^. Recently, Ibrahim et al. identified two combinations of CpGs that can accurately distinguish between CRC and normal tissues regardless of age and stage, suggesting that GSDME may be a promising biomarker for CRC detection^74^ (Table 1).

### Pyroptosis and skin cancer

GSDMC is not detectable in normal epithelial cells but is present in malignant melanoma, which may be related to the invasion and metastasis of these cancer cells^73,75^. *DFNA5/GSDME* expression in the nonresistant MeWo cell line was markedly increased compared with that in the etoposide-resistant MeWo ETO 1 cell line^76^. In etoposide-resistant melanoma cells, knockout of DFNA5/GSDME increased cell resistance to etoposide, while upregulation of DFNA5/GSDME expression increased the cell sensitivity to etoposide, suggesting that decreased-DNFA5 is related to the increase in etoposide resistance in melanoma cells^77^. Eukaryotic elongation factor-2 kinase (eEF-2K) is a negative regulator of protein synthesis that plays an important role in autophagy and pyroptosis of tumor cells under various conditions. In melanoma cells, silencing eEF-2K promoted doxorubicin-induced pyroptosis, thus sensitizing melanoma cells to doxorubicin^78^.

### Pyroptosis and gastric cancer (GC)

The NLRC4 inflammasome is involved in aseptic and autologous inflammation, and its expression in GC is higher than that in normal gastric epithelial cells^79^. In macrophages, activated-NLRC4 inflammasomes can activate caspase-1, causing pyroptosis^80^. *GSDMA* is downregulated in GC and is considered as an antioncogene^81,82^. GSDMB is not detected in most normal gastric tissue but is expressed in a few normal gastric tissue. Most precancerous samples show moderate GSDMB expression, and most cancer samples show high-level of GSDMB, which overexpression may associate with tumor invasion^82,83^. Compared with normal gastric and esophageal tissues, *GSDMC* is downregulated in GC and esophageal cancer cells and may function as a cancer suppressor gene^73,82^. In addition, the regulation of GSDML splice variant transcription and translation may alter in the gastrointestinal tract cancers^84^. GSDMD was expressed at low levels in GC cell lines and models^81,82^. Further studies showed that decreased-GSDMD regulated cell cycle-related proteins expression by activating the STAT3 and PI3K/PKB signal pathways, accelerating S/G2 phase transformation and promoting tumor cell growth. Compared with normal nude mice, the tumor volume in nude mice with low-level GSDMD was larger after the implantation of GC cells, suggesting that the GSDMD level may be related to the GC occurrence^85^. The silencing of *DFNA5/GSDME* was first reported in primary GC and GC cell lines^86^. The methylation pattern of DFNA5 was also studied in 89 primary cancer tissues, of which 52% showed abnormal DFNA5 promoter methylation^87^. Transfection of DFNA5/GSDME in these cancer cell lines decreased the number of colonies and cell growth inhibition compared to those in cells transfected with empty vector^62,88^. Moreover, the fact that p53 modulated the expression of DFNA5/GSDME strongly suggests that DFNA5 is a tumor suppressor gene^64^. Recent studies have shown that chemotherapeutic drugs can convert caspase-3-dependent apoptosis to pyroptosis through DFNA5/GSDME^89^. DFNA5/GSDME can be downregulated due to promoter methylation. Treatment with the decitabine can induce the upregulation of DFNA5/GSDME expression in tumor cells, causing pyroptosis and making these cells more sensitive to chemotherapeutic drugs^20,90^ (Table 1).

### Pyroptosis and lung cancer (LC)

In non-small cell lung cancer (NSCLC), higher GSDMD expression is related to invasive features, including more advanced tumor-node-metastasis stages and larger tumor sizes. *GSDMD*-silenced NSCLC cells show decreased epidermal growth factor receptor signaling, increased caspase 3 decomposition and enhanced apoptosis, resulting in the suppression of tumor growth in transplanted mice. Notably, the activation of NLRP3/caspase-1 signaling induces apoptosis rather than pyroptosis in tumor cells lacking GSDMD. *GSDMD*-deficiency activates the division of caspase-3 and promotes cancer cells death through the mitochondrial apoptosis pathway^91^. Xi et al. found that GSDMD contributes to cytotoxic T lymphocytes-mediated killing in lung squamous cell carcinoma and lung adenocarcinoma^92^. Lu et al. showed that knockout of DFNA5/GSDME can induce the apoptosis-to-pyroptosis switch, supporting the notion that the DFNA5/GSDME level determines the death mode of caspase-3-activated cells. In LC, loss of the *DFNA5/GSDME* gene promotes drug resistance, while overexpression of DFNA5/GSDME leads to increased drug sensitivity^93^ (Table 1).

### Pyroptosis and cervical cancer (CC)

Studies shown that the NLRP3 inflammasome participates in the innate immune response to CC and its expression is widely present in tumor cells^94,95^. NLRP3 inflammasome activation can be achieved via a half-ion channel, lysosomal rupture and reactive oxygen species (ROS). In CC, the NLRP3 inflammasome is mainly activated by the ROS to induce the pyroptosis^95^. In HPV-infected CC cells, AIM2 can play a tumor inhibitory role by stimulating pyroptosis^96^. CC cells release more IL-18 and IL-1β than normal cervical epithelial cells^97^. However, some studies have found that the removal of proinflammatory factors produced by pyroptosis can inhibit the growth of CC cells and simultaneously weaken the bodily immune effect on tumor cells^98–102^. Pyroptosis has a dual effect of promoting and inhibiting CC, but the mechanism of proinflammatory factors produced by pyroptosis in CC cells remains to be studied.

### Pyroptosis and other cancers

Nadatani et al. reported that the expression of pro-IL-18, pro-IL-1β, and NLRP3 in Barrett’s esophageal cancer cells treated with LPS was increased, and the levels of mitochondrial ROS, caspase-1, IL-18, IL-1β, lactate dehydrogenase (LDH), and pyroptosis were also increased. These results indicated that the NLRP3 inflammasome activation of caspase-1 induces the secretion of proinflammatory factors and pyroptosis. The application of a caspase-1 inhibitor can interfere with the expression of NLRP3 and block the production of IL-1β and IL-18 and the release of LDH induced by LPS^103^. In addition, the level of GSDME was more highly in esophageal squamous cell carcinoma (ESCC) than in normal adjacent tissues^104^. miR-214 suppresses cell growth and metastasis by modulating caspase-1-mediated cell pyroptosis in glioma cells^105^. A study reported that the level of MST1 is decreased in pancreatic ductal adenocarcinoma (PDAC) cells, and the restored expression of mammalian STE20-like kinase 1 (MST1) promotes PDAC cells death and suppres the proliferation, migration and invasion of PDAC cells via pyroptosis mediated by ROS^106^.

## Manipulating pyroptosis for therapeutic benefit

Pyroptosis is closely related to many human diseases^6,107–109^, including tumors^56^. In recent years, researchers have attempted to combine pyroptosis with various tumor treatments and to treat tumors by regulating pyroptosis and inhibiting the proliferation, migration and invasion of tumor cells.

### Drug-regulated pyroptosis of tumor cells

**Fig. 3:**
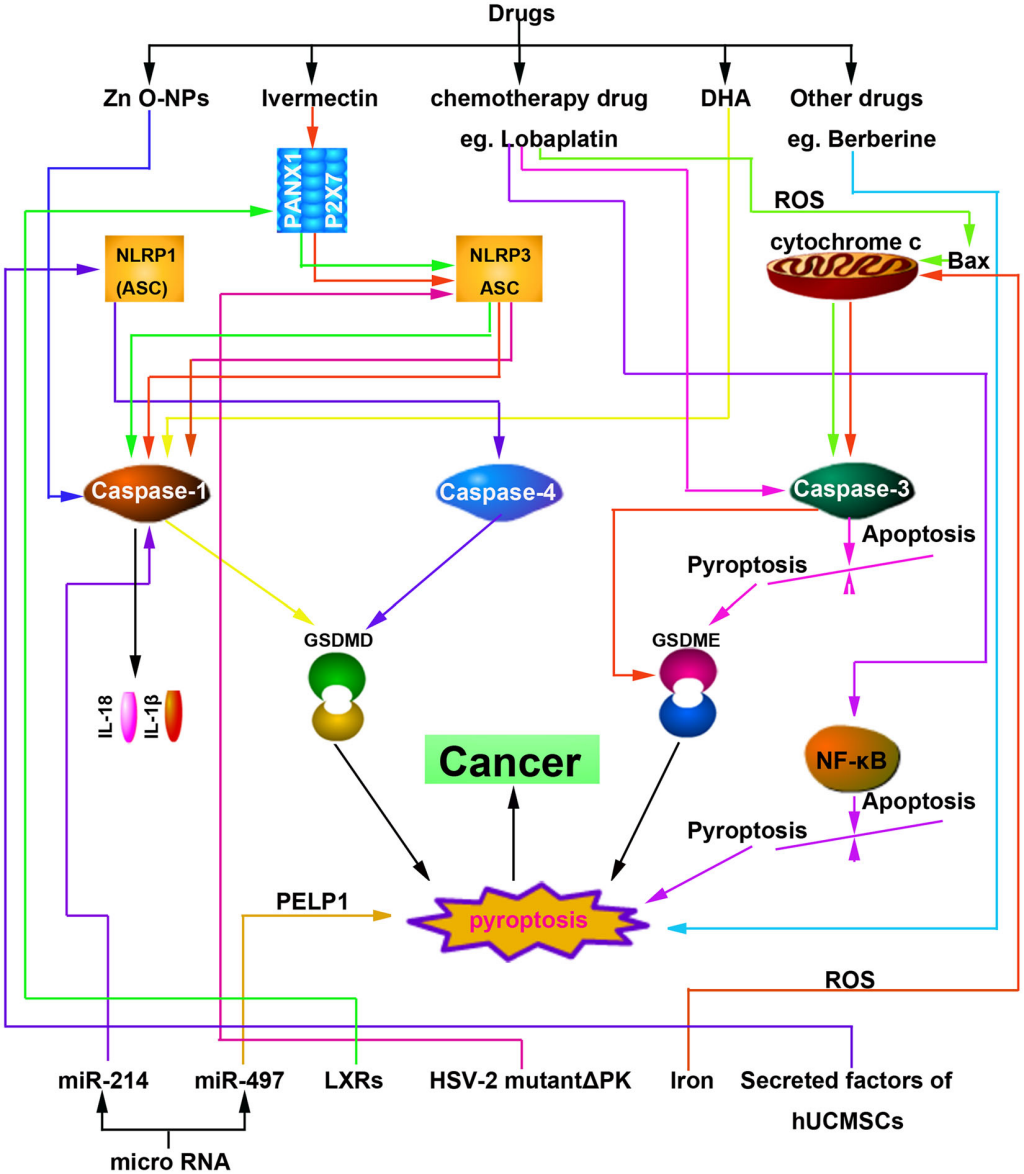
Therapeutic targets of pyroptosis core proteins in cancer. Drugs (chemotherapy drugs, new material, traditional Chinese medicines, etc.), miRNAs, receptor proteins, secreted factors of human umbilical cord mesenchymal stem cells, etc. can all modulate pyroptosis target core proteins in canonical, noncanonical, and other pathways for therapeutic benefit

Song et al. found that the level of IL-1β, caspase-1, and LDH were positively correlated with dose and time after treating A549 cells with Zinc oxide nanoparticles (Zn O-NPs), suggesting that Zn O-NPs can activate pyroptosis in A549 cells^110^. Ivermectin selectively inhibits the growth of T cell factor (TCF)-dependent transplanted tumors^111^. Dobrin et al. treated the tri-negative BC cells with ivermectin and found that ivermectin could activate the pannexin-1 channel and induce the overexpression of P2X4/P2X7 receptor. The overexpression of P2X7 receptors can release ATP, thereby enhancing the cytotoxicity caused by ivermectin, which eventually resulting in the apoptosis, necrosis or pyroptosis of BC cells^112^. In LC treatment, the thiopyran derivative L61H10 has good antitumor activity through an apoptosis-to-pyroptosis switch^113^. Nathalia et al. found that omega-3 docosahexaenoic acid can induce the pyroptosis of tri-negative BC cells and the caspase-1 inhibitor can protect BC cells from omega-3 docosahexaenoic acid-induced-pyroptosis^114^. Chu et al. found that berberine can reduce the viability and invasiveness of cancer cells by inducing the pyroptosis of liver cancer cells^56^. Anthocyanin can accelerate the death of oral squamous cell carcinoma cells by inducing pyroptosis and inhibiting tumor progression^115^. RIG-I agonists activate the pyroptosis of tumor cells, induce the expression of inflammatory cytokines, recruit leukocyte chemokines, introduce leukocytes into the tumor microenvironment, and reduce the growth and metastasis of tumors^116^. Val-boroPro can activate the ‘inflammasome’ sensor protein CARD8, which successively activates procaspase-1 to mediate pyroptosis in primary acute myeloid leukemia (AML) samples and most AML cell lines, suggesting that Val-boroPro-induced-pyroptosis is suitable for the treatment of AML^117,118^. Recently, 13d, a derivative of EF24, has been shown to be a potent antitumor agent for LC therapy that functions via the apoptosis-to-pyroptosis switch^119^ (Fig. 3).

Wang et al. found that chemotherapy drugs induced tumor cells with high-level GSDME pyroptosis due to the caspase-3 activation^20^. In tumor cell lines with low-level GSDME, the expression of GSDME was upregulated in the corresponding cell lines after treatment with the distamine, and the sensitivity of tumor cells to chemotherapy drugs was also increased, which made these cells more prone to pyroptosis. Notably, only 1/10 of tumor cells detected by the researchers had high-level GSDME, while 3/5 of human primary normal cells were found to have high-level GSDME. These high-level GSDME cells undergo pyroptosis after chemotherapeutic drugs treatment. Researchers further confirmed that GSDME-mediated pyroptosis is likely a mechanism underlying the toxic side effects of chemotherapeutic drugs^20^. In addition, chemotherapeutic drugs were found to convert caspase-3-dependent apoptosis into pyroptosis via GSDME^20,89^. In CAC cells, lobaplatin induces pyroptosis and caspase-3/9 activation downstream of the ROS/JNK/BAX mitochondrial apoptosis pathway, which is mediated by GSDME^120^. Chen et al. reported that PTX induced pyroptosis in A549 cells is closely related to the levels of active caspase-3 and GSDME-NT. Compared with PTX, the cisplatin-induced pyroptosis of NSCLC and ESCC cells was high, suggesting that cisplatin may have additional advantages in the treatment of GSDME-overexpression cancer subtypes^104,121^. *Gsdme*^*−/−*^ mice were not affected by tissue damage or weight loss induced by chemotherapeutic drugs^20^. Intraperitoneal injection of cisplatin or 5-FU resulted in severe small intestinal injury and immune cell infiltration in *Gsdme*^+/+^ mice, whereas in *Gsdme*^−/−^ mice, the signs of tissue damage were reduced^122^. In addition, *Gsdme*^−/−^ mice showed reduced lung injury and inflammatory response to cisplatin or bleomycin^122^. These observations confirm the key role of GSDME-mediated pyroptosis in promoting the harmful effects of chemotherapy and provide new insights into cancer treatment (Fig. 3).

### miRNA-regulated tumor pyroptosis

MicroRNAs (miRNA)s are noncoding single-stranded RNA containing approximately 22 nucleotides that can regulate the expression of multiple target genes. Some miRNAs, which functions similar to those of tumor-suppressor genes, can downregulate the expression of oncogenes and inhibit the growth of tumors. Jiang et al. demonstrated that miR-214 could decrease the expression of caspase-1 and inhibit tumor proliferation, migration and invasion of glioma cells. After administration of a caspase-1 inhibitor, the above mentioned abilities of tumor cells were recovered, suggesting that miR-214 can induce the pyroptosis of glioma cells by regulating the caspase-1, thereby inhibiting tumor growth^105^. Proline-, glutamic acid- and leucine-rich protein-1 (*PELP1*), a scaffolding oncogene, is highly correlated with cancer progression and outcomes for patients with advanced ESCC. Wang et al. reported that upregulating miR-497 can downregulate PELP1 and eventually induce ESCC pyroptosis, which may serve as an alternative treatment for chemo- and radiotherapy for refractory ESCC or other cancers sharing the same pyroptosis mechanisms^123^ (Fig. 3).

### Receptor protein-mediated tumor pyroptosis

Liver X receptors (LXRs) are members of the nuclear receptor family and play a key role in the inflammatory response. Studies have shown that LXRs are expressed in many cancer tissues and participate in various anticancer mechanisms. Derangere et al. found that plentiful caspase-1 was activated after LXRβ agonist T0901317 treatment, while apoptosis-related caspase-3/8/9 were not detected^124^. Further detection showed that numerous NLRP3 inflammasomes formed, and the cell death was related to the activation of the P2X7 receptor pathway and the release of ATP/ROS. Therefore, Derangere et al. believe that the LXR ligand and LXR receptor combination can open the pannexin-1 channel and release ATP, and high extracellular ATP participates in the activation of P2X7 receptor and caspase-1, inducing the formation of NLRP3 inflammasomes and thereby promoting the nonclassical pyroptosis of CAC cells^124^. Furthermore, the genetic and pharmacological inactivation of LXR in murine bone marrow-derived macrophages enhanced the inhibitory effects of radiation therapy on tumor growth through the induction of pyroptosis and activation of the inflammatory cascade^125^ (Fig. 3).

### Other ways to regulate tumor cell pyroptosis

A recent study shown that the factors secreted from human umbilical cord mesenchymal stem cells can induce MCF7 cell pyroptosis^126^. Further experiments showed that the expression of *NLRP1* and *CAPS4* and the pathways associated with inflammation were significantly changed^126^. Colunga et al. found that calpain, caspase-7 and caspase-3 were activated in numerous malignant melanoma cells infected with the herpes simplex virus type 2 (HSV-2) mutant △PK (HSV-2 mutant △PK) to promote the oncolytic effect of ΔPK. The researchers also found that △PK could increase the level of heat shock proteins, such as Beclin-1 and H11/Hsp B8; the corresponding immunohistochemical results showed that under the effect of △PK, TNF-α was activated and participated in the formation of the NLRP3 inflammasome mediated by caspase-1, which successively led to the induction of both apoptosis and pyroptosis^127^. Zhou et al. found that Tom20, an outer mitochondrial membrane protein, can be oxidized by elevated ROS, facilitating Bax recruitment to mitochondria and stimulating caspase-3/GSDME-mediated pyroptosis after iron treatment. Iron may be a potential candidate for the treatment of melanoma because it activates ROS to induce DFNA5/GSDME-dependent pyroptosis and specifically induces high levels of DFNA5/GSDME expression in melanoma cells. Further studies have shown that the use of iron supplements in patients with iron deficiency can maximize the antitumor effect of clinical ROS-induced drugs, thereby inhibiting the growth and metastasis of xenografted melanoma cells through DFNA5/GSDME-dependent pyroptosis^128^. In addition, simvastatin exerts an antitumor effect by inducing pyroptosis in NSCLC^129^ (Fig. 3).

## Conclusion and perspectives

Pyroptosis is a type of RCD mediated by the gasdermin family, and the inflammasome plays an important role in pyroptosis^20^. The occurrence of tumor cell pyroptosis in vivo suggests the potential role of pyroptosis in the regulation of tumorigenesis. Triggering the apoptosis of cancer cells has been designed and applied to eliminate malignant cells^130^. However, because one characteristic of tumors is escaping apoptosis, inducing pyroptosis is particularly important in the treatment of antiapoptotic tumors. Based on the theories of inflammation-cancer transformation and chronic inflammation-induced cell carcinogenesis, pyroptosis, as a mode of proinflammatory death, can form a microenvironment suitable for tumor cell growth. The factors related to pyroptosis have dual mechanisms of promoting and inhibiting tumorigenesis. Further exploration of the pyroptosis mechanism in different tumor cells, as well as of the proteins related to upstream and downstream of signal pathways, can provide new ideas for the treatment of related tumors.

The newly developed tumor pyroptosis therapeutic strategy shows great potential. Numerous reports have shown that chemotherapy drugs, miRNA, etc., can induce tumor pyroptosis, thereby inhibiting the malignant progression of tumors. The gasdermin family is an important group of proteins mediating pyroptosis. However, methods for preventing excessive inflammation responses by downregulating GSDMD to avoid endotoxin shock still need further study. The methylation of DFNA5/GSDME mRNA results in lower DFNA5/GSDME expression in many types of tumor cells than in normal cells^62,74,87–89,131^, which makes activating pyroptosis difficult in most tumor cells. In the chemotherapeutic treatment of malignant tumors, appropriate chemotherapeutic drugs can be selected according to the level of DFNA5/GSDME expression, which can be upregulated in tumor cells, thereby enhancing chemotherapeutic drug sensitivity and reducing drug resistance. For example, demethylating drugs, such as distamine, are combined with chemotherapy drugs, and drug combinations will be a focus of future research. In addition, the relationship between other gasdermin family proteins and tumors should also be actively researched to provide new directions for the chemotherapeutic treatment of tumors. Therefore, more experiments and clinical trials are needed to explore the potential application of anticancer therapy based on pyroptosis.

In conclusion, increasing evidence shows that pyroptosis plays a dual antitumor and tumor-promoting role during tumor progression. With the development of pyroptosis in many fields of human diseases and the gradual analysis of its mechanism, pyroptosis, similar to apoptosis and autophagy, will have an inestimable impact on the diagnosis and treatment of cancer.
